# Biomedical and environmental applications via nanobiocatalysts and enzyme immobilization

**DOI:** 10.1186/s40001-025-02782-2

**Published:** 2025-06-21

**Authors:** Yosri A. Fahim, Waleed Mahmoud Ragab, Ibrahim W. Hasani, Ahmed M. El-Khawaga

**Affiliations:** 1 Health Sector, Faculty of Science, Galala University, Galala, 43511 Suez Egypt; 2 Anatomy and Embryology Department, Faculty of Medicine, Galala University, Galala, 43511 Suez Egypt; 3 Department of Pharmaceutics, Faculty of Pharmacy, S.P.U., M.P.U and Idlib University, Idlib, Syria

**Keywords:** Biomedical applications, Environmental applications, Nanobiocatalysts, Enzyme immobilization

## Abstract

Nanobiocatalysts have emerged as transformative tools in biomedical science, enabling precise, efficient, and sustainable enzyme-based technologies. By immobilizing enzymes onto nanostructured materials, these systems overcome major limitations, such as poor enzyme stability, limited reusability, and high production costs. There are many immobilization techniques such as adsorption, covalent bonding, encapsulation, entrapment, and cross linking with a focus on their biomedical relevance. The incorporation of nanomaterials such as magnetic nanoparticles, porous silica, carbon nanostructures, and metal–organic frameworks has significantly enhanced enzyme performance under physiological conditions. A particular emphasis is placed on biomedical applications, including targeted drug delivery, high-sensitivity biosensing, thrombolytic therapy for clot dissolution, and management of oxidative stress and inflammation. The emerging role of nanozymes engineered nanomaterials with intrinsic enzyme-like activity is also discussed for their potential in diagnostics and disease modulation. Surface functionalization strategies are addressed to improve enzyme–carrier interactions and ensure biocompatibility in clinical environments. Despite promising outcomes, key challenges remain regarding large-scale production, potential nanotoxicity, and regulatory compliance. Addressing these limitations is essential for translating laboratory findings into practical biomedical solutions. This review provides a comprehensive perspective on how nanobiocatalyst-based platforms are reshaping therapeutic and diagnostic strategies in modern healthcare.

## Article Highlights

- Nanobiocatalysts enhance the stability, reusability, and cost-efficiency of enzymes for industrial and medical applications
- Enzyme immobilization with appropriate support carriers is critical to improving biocatalyst characteristics.
- Nanobiocatalysts applications include safer drug delivery, eco-friendly pollutant cleanup, and sustainable biofuel production

## Introduction

The synergy between nanotechnology (nano-biocatalysts) and enzymes has resulted in the development of some of the most promising biomaterials via the synergistic integration of advanced nano-biotechnology. The integration of enzymes into nanotechnology is of great importance in producing nanomaterials that are rarely environmentally hazardous [1]. Enzymes are indispensable for the catalysis of chemical reactions with exceptional specificity and selectivity in mild conditions, as they are natural biocatalysts. Their ability to catalyze complex reactions without requiring severe environmental conditions and their adherence to green chemistry principles have made them a fundamental component of bioprocessing [2]. However, their widespread industrial application has been impeded by obstacles, such as enzyme instability, increased production costs, and restricted reusability [3]. 

Enzyme immobilization has become a crucial method for enhancing biocatalysts'stability, reusability, and efficiency. This procedure involves attaching enzymes to solid or semi-solid substrates, allowing them to retain their catalytic functions under diverse operational settings [4]. This technique extends the functional lifespan of enzymes and facilitates their recovery and reuse, making them economically viable for ongoing industrial applications. The growing interest in enzyme immobilization highlights its potential to advance sustainable technology in various industries, such as biofuel generation, environmental remediation, and pharmaceutical synthesis [5].

Enzymes are natural macromolecules that catalyze biological reactions with exceptional efficiency and specificity. Present in all living species, they are essential for functions including metabolism, energy production, and molecular synthesis [6]. Their functional diversity arises from their capacity to identify specific substrates and facilitate reactions at rates much above those of non-catalyzed processes. In industrial applications, enzymes are valued for their capacity to catalyze reactions in mild circumstances, diminishing the energy input and environmental effect linked to conventional chemical processes [7].

Enzymes are compatible with sustainable development objectives due to their biodegradability, biocompatibility, efficacy, and reliance on renewable resources. They are extensively utilized in various industries, such as pharmaceuticals, biofuels, food manufacturing, and specialized compounds [8]. Enzymes are suboptimal for catalysis in specific large-scale applications due to their instability, limited shelf life, and susceptibility to deactivation through various means. They are also very susceptible to various adverse process conditions (e.g., elevated temperature, severe pH) and mostly unreliable due to the myriad technical hurdles encountered during their use in industrial applications. For example, unlike traditional heterogeneous chemical catalysts, most enzymes perform better when dissolved in water in homogeneous catalysis systems. Moreover, conventional methods for enzyme recovery and use are exceedingly challenging to implement.

These constraints necessitate the development of innovative methods to leverage the potential of enzymes in industrial applications completely [9].

Initially, enzyme immobilization was developed to address issues related to enzyme reuse. However, it has since evolved into a highly effective tool for addressing other enzyme limitations [10]. Immobilization of enzymes on solid supports enhances their structural integrity and resistance to denaturation in various operational environments. Immobilized enzymes are more durable for extended use due to their ability to withstand pH, temperature, and substrate concentration fluctuations. In addition, immobilization reduces the need for consistent enzyme replenishment, which minimizes waste and reduces production costs [11].

A notable advantage of immobilization is the ease of enzyme recovery and reuse. In lengthy continuous processes, immobilized enzymes can be easily extracted from reaction mixtures, maintaining their activity across multiple cycles. This reusability is particularly beneficial in industries requiring high-throughput operations, such as biofuel manufacturing and wastewater treatment. Furthermore, immobilization increases enzyme–substrate interactions, improving reaction efficiency and product yields [12].

The selection of support material is a critical factor in the effectiveness of enzyme immobilization. An efficient support material must provide a substantial surface area for enzyme immobilization, ensuring optimal interaction between the enzyme and its substrate. It must be chemically inert, mechanically stable, and biologically compatible to maintain enzyme activity and prevent undesirable side effects [13]. Advancements in nanotechnology have enabled the development of innovative support materials, such as nanotubes, nanofibers, hybrid nanostructures, and metal–organic frameworks, which enhance the effectiveness of immobilized enzymes [14]. Nanostructured supports are advantageous due to their increased surface area-to-volume ratio, facilitating effective substrate diffusion and increasing enzyme loading capacity [15]. The compatibility of these materials with enzymes is improved, and the functionalization of specific chemical groups increases their catalytic effectiveness. The straightforward separation of immobilized enzymes by magnetic fields is facilitated by magnetic nanoparticles, which streamlines downstream processing and reduces operational expenses [16].

Immobilized enzymes have been utilized across various sectors, including biomedicine, agricultural processing, environmental remediation, and renewable energy generation. The shift to healthier energy sources is enabled by the effective conversion of biomass into bioethanol and biodiesel in biofuel production, which is enhanced by immobilized enzymes [17]. Immobilized enzymes are utilized in environmental remediation to decompose contaminants and hazardous materials, offering a sustainable alternative to traditional chemical treatments. Developing sophisticated support materials and immobilization methods enhances enzyme-based technologies'potential [18].

This review is crucial, since it presents a comprehensive analysis of these vital factors, imparting valuable insights into the design and optimization of immobilized enzyme systems. This review aims to enhance the efficiency and cost-effectiveness of biocatalytic processes by examining the problems and opportunities in this domain, thus connecting fundamental research with practical applications.

## Preparation and immobilization methods of enzymes loaded nanomaterials

### Preparation techniques of nanomaterials

**Fig. 1:**
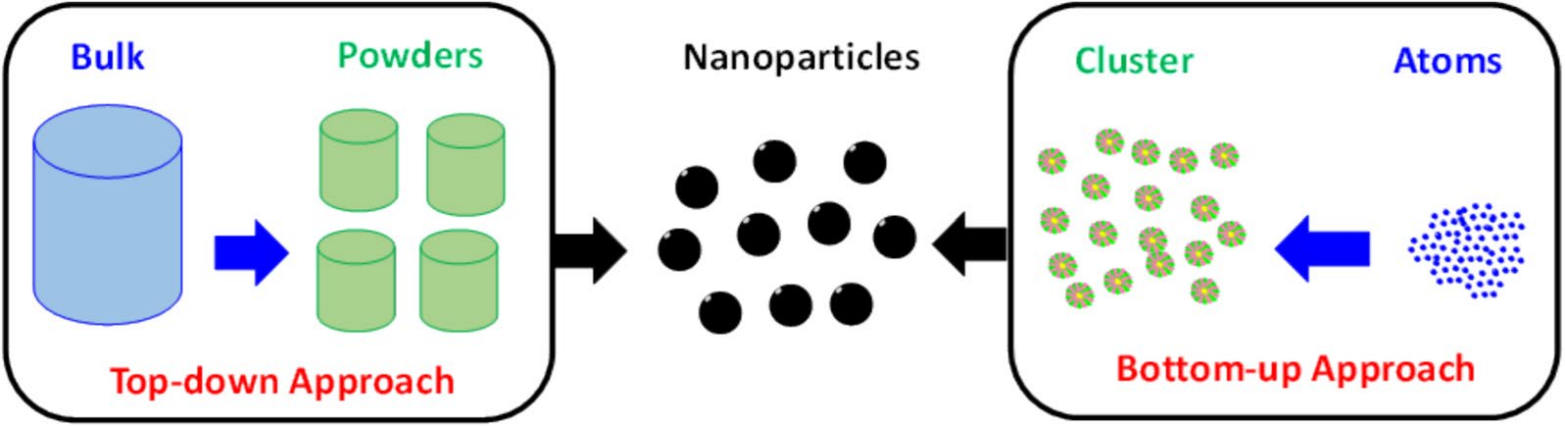
Synthesis techniques of nanoparticles

Nanomaterials may be synthesized by two principal methodologies: top-down and bottom-up procedures, as shown in Fig. 1. The top-down approach involves the fragmentation of bulk material into nanoscale particles [19]. The bottom-up technique entails the incremental construction of a material at the atomic or molecular level to create intricate nanoscale structures. Kinetic mechanisms govern the size and morphology of nanoparticles in both methods [20].

#### Top-down methods

This approach involves converting a large amount of material into very small particles, also referred to as nanosized particles. Nanoparticles are produced by reducing the size of the original material through various physical and chemical techniques [20]. These techniques includes ball milling, heat treatment, Mechanochemical synthesis, laser ablation and Ion sputtering [21].

#### Bottom-up methods

Bottom-up processes involve the synthesis of nanoparticles by combining smaller molecules, such as atoms, molecules, or microscopic particles. In this technique, the nanostructured components of the nanoparticles are first fabricated, and then assembled to form the final nanoparticle. These techniques include physical and chemical vapour deposition, Sol gel, Coprecipitation, Hydrothermal methods [22]. The advantages and disadvantages of employing top-down versus bottom-up techniques are listed in Table 1.

**Table 1:** Advantages and disadvantages of top-down and bottom-up approaches

Method	Advantages	Disadvantages
Top-down	• Enables large-scale production • Eliminates the necessity for chemical modification or treatment • Facilitates uniform distribution over extensive substrate areas	• Exhibits a broad size distribution ranging from 10 to 1000 nm, with diverse particle morphologies • Precise control over deposition parameters remains challenging • Involves a technically intricate and cost-intensive process
Bottom-up	• Cost-effective approach • Allows for the attainment of nanoparticle sizes from 1 to 20 nm • Enables the synthesis of nanoshells, ultrafine particles, and nanotubes • Permits modification of deposition conditions to suit specific requirements	• Large-scale production presents significant challenges • Chemical purification is necessary for nanoparticle refinement • The process may be overly complex and time-intensive, often involving numerous assumptions and uncertainties

### Enzyme immobilization techniques

**Fig. 2:**
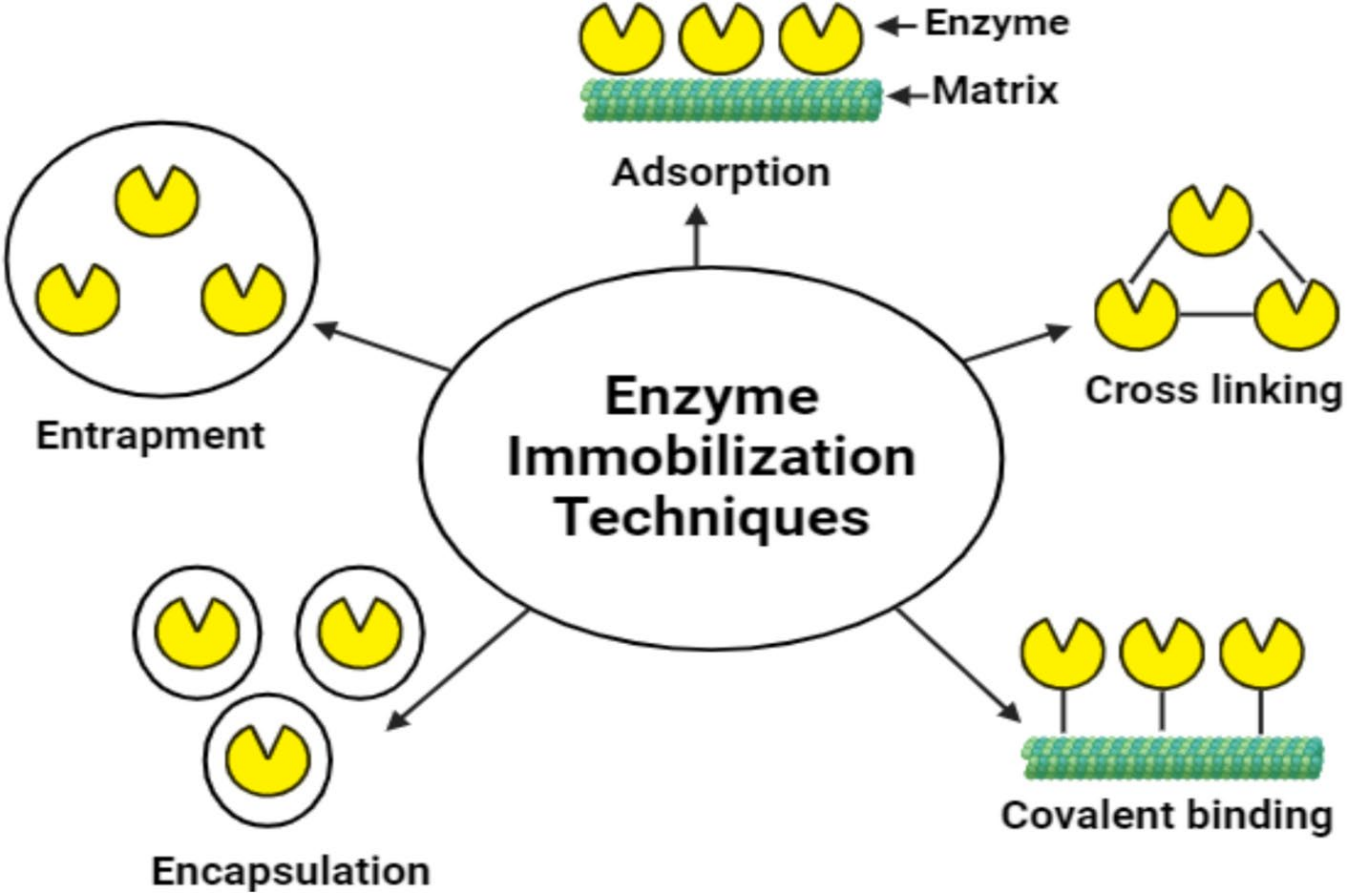
Various methods of enzyme immobilization

Enzyme immobilization is a requirement in a number of industrial applications [23]. There are many different methods used for enzyme immobilization. These approaches enhance enzyme stability by forming additional chemical bonds, limiting the movement of substrates and products with physical barriers, partially obstructing specific inhibition sites through distortion or blockage, and modifying the surrounding physicochemical environment [24]. There are five distinct categories for categorizing enzyme immobilization strategies. The primary techniques for enzyme immobilization include adsorption, covalent bonding, entrapment, encapsulation, and cross-linking, each offering distinct advantages and limitations. Adsorption is a simple and reversible method that utilizes non-covalent interactions, but often suffers from weak binding and limited reusability. Covalent bonding, by contrast, forms strong, stable links between enzymes and carriers, improving durability and reusability, though it may restrict enzyme flexibility. Entrapment involves enclosing enzymes within polymeric matrices, providing protection while allowing substrate access, but can lead to mass transfer limitations and potential enzyme leakage. Encapsulation confines enzymes within semi-permeable membranes, enhancing stability and suitability for biosensors, though it may suffer from diffusion barriers or membrane rupture. Finally, cross-linking forms enzyme aggregates without the need for external support, offering cost-effectiveness and increased resistance to harsh conditions, although harsh chemicals and limited control over enzyme orientation can impact activity. These methods are selected based on the desired stability, activity, and application of the immobilized enzymes, and are often optimized to enhance performance in biomedical and industrial systems, as illustrated in Fig. 2*.*

### Nanomaterials as support matrix for enzyme immobilization

**Fig. 3:**
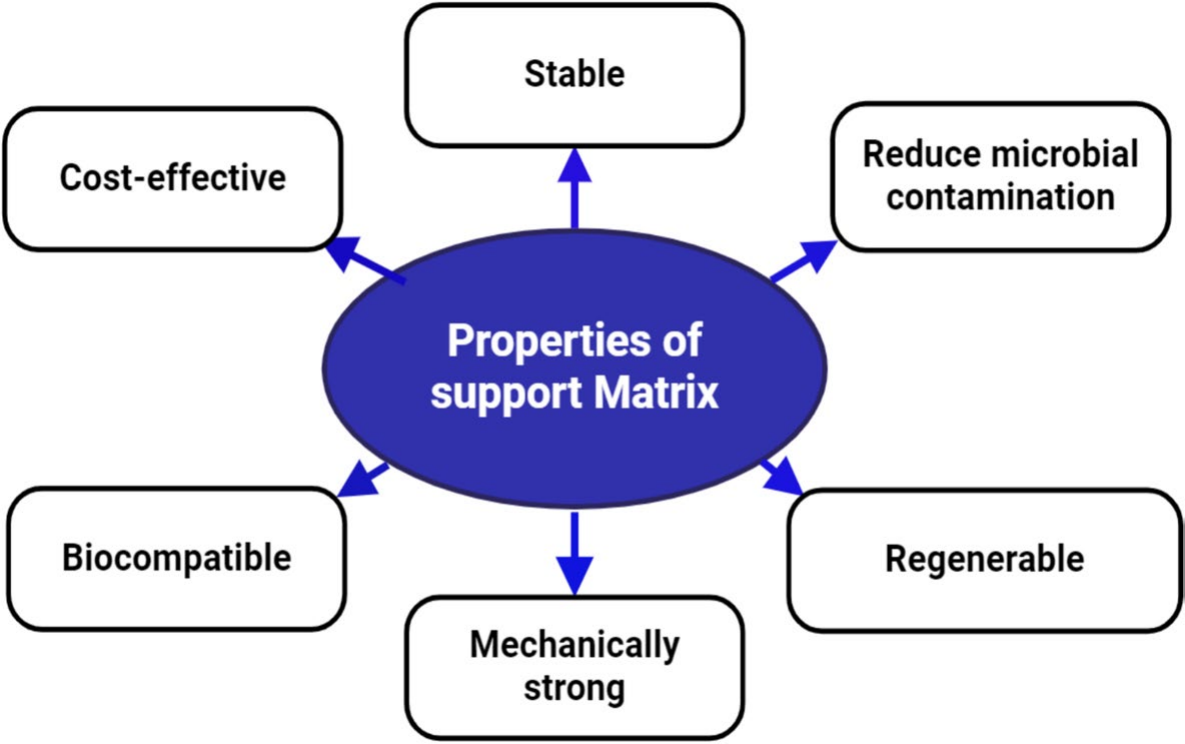
Properties of support matrix

The selection of support material is essential in enzyme immobilization, since it directly influences the immobilized enzyme's stability, activity, and reusability [25]. Support materials establish a robust framework for enzyme binding and foster an optimum microenvironment that improves enzyme efficacy. Figure 3 shows some properties of Support matrics.

#### Metal nanomaterials

Metal nanoparticles demonstrate a diverse range of catalytic properties. Significant attempts have been made to develop catalytic systems that integrate the characteristics of biocatalysts with those of metallic nanoparticles [26]. Metallic nanoparticles, such as gold, platinum, and palladium, are ideal for this application due to their small size, large surface area, and distinctive quantum properties [27, 28]. These nanomaterials can enhance the flow of electric charge, distribute enzymes, and regulate enzymatic activity [29].

#### Porous silica and carbon-based nanomaterials 

Encapsulating enzymes within porous silica structures can greatly improve their stability and selectivity [30]. This confinement also enables the isolation and reuse of enzymes. Moreover, porous silica materials have shown promise as carriers for enzyme immobilization [31]. Carbon-based nanostructures possess unique characteristics, including a significant surface area, exceptional chemical stability, and remarkable electrical and thermal properties [32]. These attributes make them ideal for enhancing materials in the modification of biocatalysis [33]. Carbon materials have enhanced charge transfer, modified enzyme function via photothermal effects, and modulated enzyme activity employing photosensitive compounds [34].

#### Magnetic nanoparticles

Magnetic nanoparticles (MNPs) are commonly utilized for protein and enzyme immobilization due to their unique properties, including easy separation in a magnetic field, high surface-to-volume ratio, superparamagnetic characteristics, exceptional reusability, low toxicity, large surface area, significant enzyme capacity, and the ability to modify surface surfaces through chemical reactions [35, 36]. These properties substantially enhance the capacity for enzyme loading and diminish diffusion limitations. The magnetic bio-separation technique efficiently isolates a target product from enzyme-immobilized magnetic particles utilizing a primary magnet [37]. Moreover, numerous studies have shown that immobilizing enzymes on nanoparticles reduces protein unfolding while enhancing stability and effectiveness [38].

#### Nanofiber

Nanostructured fibers (NFs) exhibit exceptional properties, such as a high enzyme loading capacity and a consistent distribution in the liquid phase [39]. Furthermore, these structures'high porosity and interconnectedness result in minimal resistance to mass transfer. The distinctive characteristics of nanostructured fibers (NFs), including their surface properties, nanostructures, and self-assembly capabilities, present promising opportunities for optimizing bioprocesses in bioreactor systems [40]. A compelling assembly was created by integrating enzymes with nanostructured fibers (NFs), yielding a remarkable network that unites the superior biocatalytic features of enzymes with the distinctive characteristics of NFs [41].

#### Organic nanomaterials

Organic nanomaterials, such as cellulose or starch nanoparticles and chitin or chitosan nanofibers, have been extensively studied for their unique properties. Unlike their larger-scale counterparts, these nanofibers exhibit excellent enzyme-binding capabilities and uniform dispersion [42]. They offer high porosity, reduced mass transfer resistance, and the ability to prevent protein denaturation and enzyme dysfunction. Because of these traits, nanofibers are often utilized as carriers for enzyme immobilization. Recently, bacterial cellulose nanofibers have been used to immobilize lysozyme [43]. In addition, combining organic and inorganic materials has led to the development of hybrid nanomaterial supports with exceptional characteristics, surpassing those of their individual components. These hybrid supports enhance enzyme stability, enable recycling, and protect enzymes from denaturation, maintaining their biological activity during reactions [44]. For instance, magnetic nanoparticles combined with organic polymers, such as acrylamide, cellulose, or chitosan, form core–shell structures [45].

#### Metal–organic frameworks (MOFs)

Metal–organic frameworks (MOFs) effectively support enzyme immobilization due to their porous topologies, broad surface areas, and adaptability [46]. These crystalline materials, composed of metal ions and organic ligands, provide a protective environment for enzymes, enhancing their stability under adverse conditions, such as high pH, temperature, and organic solvents. MOFs'modular nature allows for precise customization, which increases enzyme loading, activity, and reusability [47]. This renders MOFs more advantageous than conventional supports, such as polymers and silica. Multiple methods of enzyme immobilization on MOFs include physical adsorption, chemical bonding, diffusion, and in situ encapsulation [48]. MOF–enzyme composites have the potential to be used in biomedicine as therapeutic nanoreactors and for targeted drug delivery, as they can protect enzymes from degradation and immunological reactions.

### Surface modification of nanobiocatalysts and factors affecting immobilized enzymes

Different chemical functional groups, such as –NH_2_, alcoholic ^–^OH, ^–^COOH, and ^–^SH, can be attached to the material's surface, enabling covalent interactions with enzymes under specific conditions [49]. Post-functionalization generally entails the activation of supports using certain agents, including organic and inorganic halides, glutaraldehyde, carbodiimides, and other bifunctional agents, to promote enzyme immobilization [50]. Functionalization or derivatization denotes the process of incorporating a new chemical function onto a support (enzyme carrier), whereas activation guarantees that the newly introduced chemical group becomes reactive towards the enzyme. These steps may transpire concurrently. Enzymes can be immobilized on functionalized magnetic nanoparticles through various techniques, including magnetic attraction, covalent bonding, or physical adsorption [51]. Magnetic nanoparticles effectively isolate mounted enzymes from the reaction mixture with the use of an external magnetic field. Magnetic nanoparticles are often modified with organic polymers, which can be either synthetic or biopolymers. These polymers include functional groups on the surface, creating binding sites for enzymes. A key benefit of using polymers is their capacity to integrate monomers and associated functional groups selectively for enzyme immobilization. Modifying the surfaces of magnetic nanoparticles with functional groups through organic polymers can improve interactions between the support and the enzyme, as well as between the enzyme and substrate, ultimately enhancing enzymatic activity [52]. The immobilized enzyme system's durability is essential for long-term use, particularly in continuous processes. In the event of mechanical degradation of the support material, enzyme leakage or activity loss may occur. Although there are significant advantages, it is also essential to recognize the constraints related to immobilized enzymes, as detailed in Table 2.

**Table 2:** Advantages and disadvantages of enzymes immobilization

Advantages	Disadvantages
• Enzymes can be repeatedly utilized, hence reducing the overall cost of the process • Immobilized enzymes are easily removed from the reaction mixture, facilitating downstream processing • Immobilized enzymes facilitate simpler addition and removal, enhancing process control • Immobilization diminishes enzyme contamination in the final product • Immobilized enzymes are better suited for widespread industrial applications • Immobilized enzymes can be utilized in continuous flow systems, enhancing production	• Some enzymes may lose their catalytic activity during the immobilization process • The availability of substrate to the enzyme may be constrained, diminishing reaction efficiency • The operation may be technically challenging and time intensive • Due to structural or functional restrictions, not all enzymes can be immobilized • The selection of carrier material significantly influences enzyme activity • Enzymes may detach from their carrier over time, reducing efficiency

### Parameters affecting immobilized enzymes

**Fig. 4:**
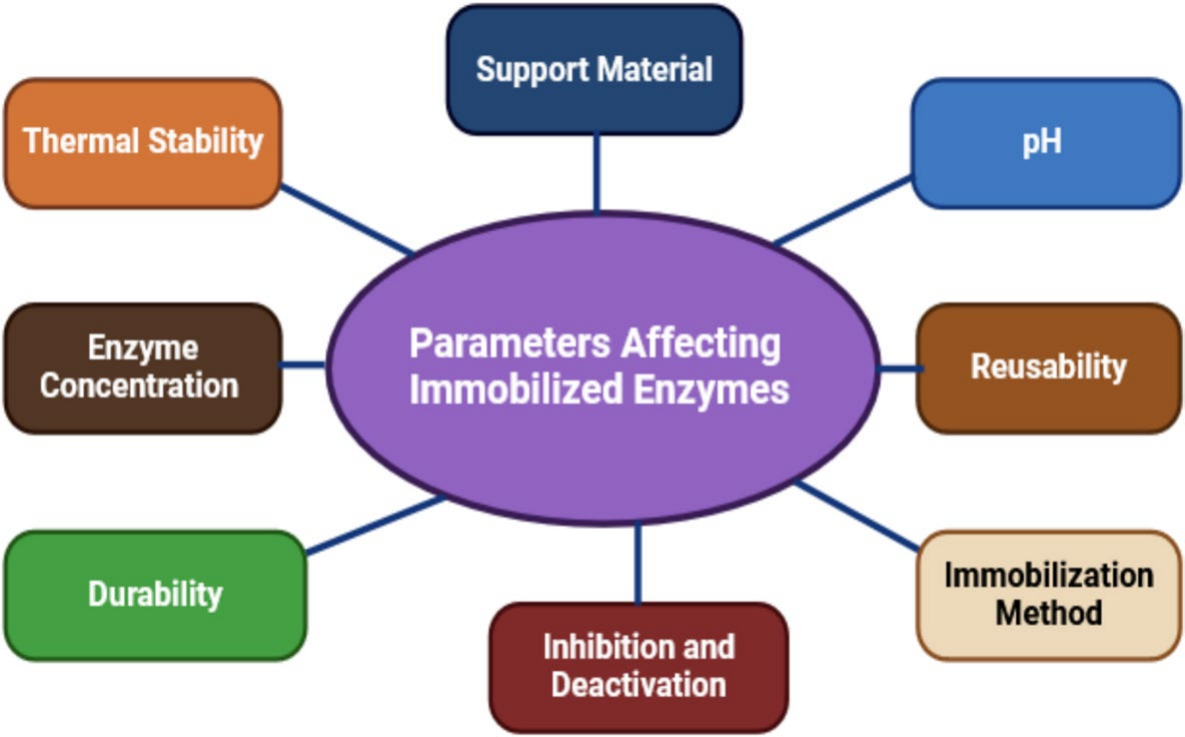
Parameters affecting immobilized enzymes

The performance of immobilized enzymes is influenced by several key factors, Fig. 4. These factors collectively determine the efficiency, stability, and applicability of immobilized enzymes in various industrial and biomedical contexts:

- *Support material compatibility*: The support must be chemically compatible with the enzyme and reaction conditions to prevent denaturation or degradation. Surface chemistry, including functional groups, affects enzyme binding, conformation, and stability.
- *Hydrophobicity/hydrophilicity*: The support’s surface properties influence enzyme orientation, substrate accessibility, and activity.
- *Pore size*: Optimal pore size ensures efficient mass transfer and sufficient surface area for enzyme attachment.
- *Immobilization method*: Techniques such as adsorption, covalent bonding, entrapment, or encapsulation are chosen based on stability, enzyme loading, and application needs, each with specific benefits and limitations.
- *pH and temperature*: Immobilization can alter the enzyme’s optimal pH and enhance thermal stability, allowing functionality at higher temperatures or extreme pH levels compared to free enzymes.
- *Enzyme concentration*: The amount of enzyme and its loading on the support impact immobilization efficiency and activity, requiring optimization.
- *Inhibitors*: Molecules present during immobilization can deactivate enzymes, necessitating careful selection of compatible substances.
- *Reusability and stability*: Strong enzyme-support interactions, such as covalent bonding, enhance reusability by reducing enzyme leaching. Mechanical stability of the support is critical to prevent enzyme loss during continuous processes.

## Biomedical applications of nanobiocatalysis

Nanobiocatalysis arises from the combination of nanotechnology and biotechnology. Various techniques utilized to encapsulate natural enzymes onto nanostructures effectively have resulted in the creation of nanobiocatalysts with significant promise in applications, such as the food industry, wastewater treatment, biofuel generation, and medical diagnostics.

### Drug delivery systems

Endogenous enzymes are crucial in the microenvironment of particular tissues, facilitating the regulated and exact release of pharmaceuticals. This contributes to the effective management of various diseases [53]. Enzymes engage with nanocarriers to augment processes within the host system. Hydrolase enzymes, including lipases, proteases, and glycosidases, are extensively utilized in drug delivery applications [54]. This technique enables the efficient and controlled release of drugs and is referred to as a hydrolase-responsive nanomaterial. Enzyme-activated drug transporters play a crucial role in improving the targeted delivery of therapeutic agents to specific regions and facilitating their uptake by particular cells [55, 56]. Drug release from diverse nanocarriers transpires via enzymatic breakdown at designated locations. Nanomaterials, such as crosslinked matrices, self-assembled systems, and caged porous structures, can encapsulate pharmaceuticals by either physically entrapping them or by chemical bonding [57]. Biological enzymes, such as glycosidases, phosphatases, ureases, and amidases, are crucial for facilitating the directed delivery of pharmaceuticals to the infection site [58]. Certain enzymes, such as glycosidases, can hydrolyze complex polysaccharides into simpler saccharide units [59]. This process occurs when these enzymes are released in the vicinity of the tissue they are meant to target. While various nanocarriers demonstrate different drug release rates, researchers are actively developing methods to improve the specificity of these nanostructures for targeted body regions [60]. In addition, researchers are investigating ways to minimize immune responses by chemically modifying or coating the nanocarriers with compounds, such as polysaccharides and peptides. To ensure precise and effective distribution to designated locations, it is crucial to account for several environmental parameters, such as pH, concentration, viscosity, and toxicity levels. A thorough understanding of the required doses and methods is essential for the safe development and use of nanoparticles in drug delivery [61].

### Biosensing applications

Nanomaterials serve as optimal substrates for biomolecule immobilization due to their ease of surface modification. The remarkable properties of nanomaterials have been extensively utilized to create various biosensors employing techniques, such as electrochemistry, colorimetry, fluorescence, and surface plasmon resonance (SPR) [62]. A biological sensing element and a detector are necessary for biosensing systems that employ nanobiocatalysts to identify and detect an analyte [63]. This detector transforms the physicochemical alterations induced by the biological component into a quantifiable signal that correlates directly with the analyte's concentration. Enzymes are the primary biological components used in the construction of biosensors [64]. A suspension-based assay was developed to measure uric acid by adding a sample solution to the suspension containing micro-sized particles immobilized on uricase and horseradish peroxidase (HRP) [65]. Enzyme-based biosensors typically offer high sensitivity, rapid response times, resistance to electrical and magnetic interference, structural versatility, and the ability to be miniaturized to micro- and nanoscale dimensions [66]. They are also cost-effective, lightweight, and can be alternatives to bulky laboratory-based sensing equipment. Typically, nanoparticles have a large surface area at the material interface, allowing them to bind enzymes with a high loading capacity, which helps stabilize the enzyme by maintaining its structural integrity [67].

In addition, the ability to modify nanomaterials holds considerable potential for enhancing the performance of sensors that depend on nanoelectronics [68]. Moreover, nanoparticles made of noble metals such as gold, silver, and platinum can substantially improve the electrical conductivity of the enzyme layer affixed to the electrode [69]. This leads to improved sensitivity and swift detection in electrochemical biosensors. Noble metal nanoparticles function as catalysts in electrochemical processes, particularly in the redox catalysis of H2O2, as demonstrated by the development of glucose biosensors utilizing gold nanoparticles [70]. Iron oxides and other nanomaterials can serve as electrocatalytic substrates for enzyme attachment, enabling the development of various biosensors [71].

### Thrombolytic therapy

Enzymes such as tissue plasminogen activator (tPA), streptokinase, and urokinase-type plasminogen activator (uPA) serve as therapeutic treatments to inhibit coagulation in instances of acute myocardial infarctions or small cerebral thrombi [72]. Nonetheless, non-targeted stimulation may result in inadvertent and excessive hemorrhaging. Magnetic nanoparticles, liposomes, and polymeric nanoparticles have been used alongside thrombolytic enzymes to alleviate these limitations [73]. This combination allows for targeted intervention at the site of blood clot formation, reducing associated risks. Nanoparticles can deliver thrombolytic enzymes directly to areas, where blood clotting occurs, minimizing the likelihood of adverse effects [74]. Empirical data demonstrates the efficacy of magnetic nanocarriers in transporting streptokinase to the locus of canine carotid artery thrombosis under the influence of an external magnetic field. Mesoporous nanoparticles are very efficacious in facilitating the breakdown of blood clots [73]. This property makes them an ideal material for significantly increasing the loading capacity of urokinase by up to 30 times [75].

### Treatment of oxidative stress and inflammation

Cells commonly use catalytic processes involving enzymes such as peroxidases, superoxide dismutase (SOD), and catalase to neutralize internal reactive oxygen species (ROS). Administering external SOD and catalase directly to the site of inflammation can effectively mitigate the excessive production of ROS [76]. Polyethylene glycol (PEG) and poly(lactic–co-glycolic acid) (PLGA) copolymers, in conjunction with oleate-coated magnetite nanoparticles, served as nanocarriers to protect enzymes from degradation during injection, owing to their generally restricted stability [61]. Polymeric nanoparticles loaded with catalase or SOD have been shown to quickly reach the pulmonary vasculature within 30 min of intravenous administration, with a 33% success rate. In addition, these particles can provide protection against acute inflammatory responses triggered by endotoxin exposure in mice [77].

### Roles of nanozymes in biological functions

Nanozymes, or nanomaterials with enzyme-like catalytic functions, have become crucial in biological systems for mimicking natural enzymes, such as peroxidase, oxidase, catalase, and superoxide dismutase (SOD). These materials catalyze reactions involving reactive oxygen species (ROS) and hydrogen peroxide (H₂O₂), playing key roles in cellular signaling, oxidative stress modulation, and immune responses [78]. Their superior stability, resistance to harsh physiological conditions, and customizable properties achieved through modifications in size, shape, and surface chemistry make them ideal candidates for a wide range of biomedical applications. Therapeutically, nanozymes function as both active agents and precision delivery systems. For instance, cerium oxide nanozymes can cycle between oxidation states to neutralize ROS, reducing damage in conditions, such as inflammation and neurodegeneration [79]. In oncology, catalase- or peroxidase-mimicking nanozymes modulate the tumor microenvironment, enhancing immune cell activity and promoting immunogenic cell death (ICD), thereby improving responses to immunotherapies [80]. Diagnostically, nanozymes are integrated into biosensors to detect biomarkers through catalytic colorimetric or electrochemical signals. Their robustness, low cost, and reusability give them an edge over natural enzymes, bridging the gap between nanotechnology and modern medicine in tackling complex biological challenges [81].

### Other industrial, agricultural and environmental applications of nanobiocatalysts

#### Food and agricultural industries

Immobilized enzymes are utilized extensively in the food industry for various reasons, such as their exceptional thermostability and endurance during long-term food processing [82]. Enzymes employed in the food business were immobilized by various support materials and techniques. Protease was immobilized via cross-linking with chitosan and employed to eliminate gluten from the beverage. Due to immobilization, the enzyme significantly diminished the gluten concentration in the original beer from 65 mg/kg to 15 mg/kg following 10 h of treatment [83]. Covalently immobilized amylase with chitosan increased the conjugate's thermal stability by 35%, strengthened the amylase's resistance to pH inactivation, and increased the product yield of barley hydrolysis by 1.5 times [84]. Immobilized lipase enzymes are commonly utilized in the dairy sector to improve cheese flavor through fat hydrolysis and to accelerate the maturation process. Moreover, they are utilized in the lipolysis of butterfat [85]. α-Lactalbumin nanotubes served as carriers for lipase, producing quantities of free fatty acids that were much higher (1.5-fold) than those of the free enzymes. The hydrolysis of lactose in dairy products is a common application for β-galactosidase [86].

Nanotechnology is progressively employed in agriculture for several applications, including enhancing crop growth, improving soil quality, and developing more efficient and targeted pesticide delivery systems. Conventional fertilizers are sprayed indiscriminately in agriculture to meet the increasing food demand from a growing population, since these chemical fertilizers demonstrate inadequate nutrient absorption and substantial losses. Tomato plants subjected to Cu NPs at 250 mg L^−1^ exhibited enhanced fruit quality and bioactive chemicals, but treatment at 500 mg L − 1 adversely affected the bioactive compounds in tomato fruit [87]. Nevertheless, the potential environmental and health risks associated with the use of nanotechnology in agricultural products are a cause for concern. To guarantee the secure application of nanotechnology in agricultural products, regulatory agencies worldwide have established policies and regulations. The following are some critical regulations and guidelines for agricultural products that are based on nanotechnology.

- *Regulatory oversight*: The application of nanotechnology in agriculture is governed by the regulations of each country. In the European Union, nanotechnology in foods and pesticides is regulated by the European Chemicals Agency (ECHA) and the European Food Safety Authority (EFSA).
- *Risk assessment*: Regulatory bodies conduct risk studies prior to the licensing of nanotechnology-based agricultural products. This involves assessing the toxicity of the utilized nanomaterials, the likelihood of environmental dispersion, and the implications for human health.
- *Labeling*: Regulatory agencies mandate the labeling of agricultural goods that use nanomaterials. This empowers consumers to make educated choices regarding their purchases and usage.
- The safety and quality of agricultural products that are founded on nanotechnology are guaranteed by international standards.
- *Research and development*: Regulatory agencies advocate research and development to ensure the safety of nanotechnology-derived agricultural products for human health and the environment. The secure application of nanotechnology in agricultural products necessitates a collaborative endeavor among customers, regulatory bodies, manufacturers, and researchers.

#### Wastewater treatments and environmental decontaminations

Effluents or wastewater from various industrial processes, including textiles, papermaking, tanning, and printing, are known sources of dyes and colorants. These substances are considered carcinogenic and hazardous, even in small amounts [88]. Heavy metals such as lead (Pb), cadmium (Cd), chromium (Cr), and arsenic (As) present serious public health risks due to their toxicity and environmental persistence [4, 89]. Enzymes such as laccases, peroxidases, and lipases can be used to treat these effluents, especially those containing fatty wastes [90]. The stability and activity of nanobiocatalysts can be affected by the pH, ionic strength, and other conditions that can be altered by the presence of additional molecules in wastewater or detritus [91]. The extreme physical and chemical conditions commonly seen in effluent streams can disrupt the normal conformation of enzymes, resulting in a decrease in their efficacy and loss of catalytic activity [92]. Enzyme activity loss is reduced during operation by immobilizing enzymes on solid supports, allowing for the reuse of the biological component. For example, the catalytic efficiency and stability of horseradish peroxidase (HRP) were preserved when it was immobilized onto magnetic nanoparticles [93]. This facilitated the efficient decolorization of azo-dyes. In addition, persistent contaminants in wastewater were tackled through the application of oxidative enzymes, especially laccases, which were immobilized onto carbon nanotubes [94]. Applying enzymes immobilized on nanoparticles (NPs) for wastewater treatment is notably advantageous, because they decompose pollutants into less deleterious by-products [95]. Enhancements in wastewater treatment can be achieved by integrating knowledge from protein chemistry, biochemistry, and nanotechnology to create single enzyme nanoparticles (SENs) for purifying purposes [96]. In environmental applications, it is essential to ensure that Nanoenzymes (NEs) remain stable even under harsh conditions. In addition, NEs have been widely used to purify drinking water due to their low toxicity and excellent biodegradability [97].

#### Biofuel production

The growing urgency to tackle environmental challenges and the depletion of fossil fuels has significantly driven the development of a green and sustainable bioprocessing approach for biofuel production through enzymatic technology [98]. Biofuels produced through bioconversion, such as biodiesel, bioethanol, biohydrogen, and biogas, are environmentally sustainable and can be replenished indefinitely [99]. Enzymes have been used as alternatives to conventional chemical catalysts. For example, the production of biodiesel through lipase-based bioprocesses offers a more energy-efficient and environmentally sustainable method compared to traditional alkaline-catalyzed processes [100]. Another significant example is the use of enzymes to accelerate the breakdown of cellulose into fermentable sugars, which can subsequently be utilized to produce bioethanol [101]. Biodiesel is traditionally produced through chemical processes involving organic solvents and high temperatures. Nanocarriers have shown the ability to create chemical and thermal-resistant nanobiocatalysts, offering enhanced enzyme capacity and superior catalytic activity [102].

#### Detergent industry

Enzymes have long been used in detergent compositions to remove specific stains that conventional detergents cannot reach. Enzyme-based detergents can also be used in tiny doses because of their ability to remove stains at room temperature. For example, proteases are required to eliminate blood, fish, egg, meat, and grass strains. In addition, they may effectively remove the protein types found in human perspiration. Conversely, amylases are used in detergent formulas to eliminate starch stains from gravies, cereals, potatoes, and chocolates. Lipases are good at removing oil and fat stains. In addition to stain removal, enzyme-based detergents have other uses. For example, cellulases improve the softening and color enhancement of cotton-based materials [103]. The immobilization of detergent enzymes enhances cleaning performance, preserves the enzymes'catalytic activity, and is not harmful to wool fibers. Proteases hydrolyze natural protein fibers, including silk and wool keratins, causing permanent degradation of the garment's quality. Lipases constitute the second most essential category of detergent enzymes, subsequent to proteases [104]. Lipases are included in laundry and dishwashing detergents to eliminate oil stains. They function well across a range of temperatures and pH levels [105]. Nevertheless, lipases exhibit limited efficacy in cleaning with regular water due to mass transfer impediments between the lipids and the enzymes [106]. Lipases were immobilized using adsorption and cross-linking with glutaraldehyde, and the experiment on woolen fabric demonstrated remarkable oil stain removal effectiveness, sustaining 80% catalytic activity over the washing cycle [107]. Table 3 presents a list of different applications for enzymes immobilized on different nanomaterials.

**Table 3:** Different immobilized enzymes and their applications

Nanomaterial	Immobilized Enzyme	Applications	Ref
Au–chitosan NPs	Peroxidase	Biosensor for hydrogen peroxide in water, pharmaceutical, and biomedical applications	[108]
Se NPs	Glucose oxidase	Biosensor for the quantification of glucose in biological fluids, food items, and agricultural products	[109]
ZnO NPs	Uricase	Uric acid biosensor in serum	[110]
Au NPs	Glucose oxidase	Catalytic nanodevice for the construction of nanoreactors	[111]
NiO NPs	Glucose oxidase	Amperometric biosensor for glucose	[112]
Cu NPs	Tissue plasminogen activator, streptokinase	Reestablishes blood circulation in arterial thrombosis	[113]
Silver NPs	Lysozyme	Antibacterial efficacy against diverse resistant bacterial strains	[114]
Alumina nanoparticles	Streptokinase	Thrombolytic colloid exhibiting extended efficacy	[115]
Chitosan NPs	Urokinase	Enhanced thrombolytic activity	[116]
Cu (II)-chelated chitosan NPs	Laccase	Degradation of phenolic compounds	[117]
Fe_3_O_4_/nanotubes	Horseradish peroxidase	Removal of phenols from wastewater	[118]
Titania NPs	Laccases	Biotransformation of contaminants, such as diclofenac and acetaminophen in aquifers	[119]
ZnO nanoparticles	Lipase	Requested the elimination of oil and grease stains from cotton fibers	[120]
Silica nanoparticles	*α*-Amylase	Improved cleaning efficacy for starch elimination on cotton textiles	[121]
Silica nanoparticles	Protease	Demonstrated enhanced cleaning efficacy for the removal of protein dirt from cotton fibers	[122]
Iron oxide/chitosan nanocomposite	Manganese peroxidase	Discoloration of textile wastewater	[123]
Fe_3_O_4_ NPs	Catalase	Decomposition of hydrogen peroxide	[124]
Graphene oxide nano powder	Horseradish peroxidase	Adsorption of methylene blue from aqueous solutions	[125]
TiO_2_NPs	*α*-Amylase	Starch hydrolysis	[126]
Ag NPs	*β*-Galactosidase	Lactose hydrolysis	[127]
Au nanorods	*α*-Amylase	Starch hydrolysis	[128]
Nickel NPs	Diastase	Starch hydrolysis	[129]
SiO_2_ NPs	*β*-Glucosidase	Sugarcane juice treatment to increase phenolics	[130]
Nanocellulose/polypyrrole/GO	Lipase	Synthesis of flavors	[131]
Fe_3_O_4_-chitosan NPs	*β*-Galactosidase	Galactooligosaccharides production	[132]
zinc oxide NPs	β-Galactosidase	Lactose hydrolysis	[133]
Ferric silica NCs	Lipase	Biodiesel production	[134]
silver NPs	*α*-Amylase	Starch hydrolysis	[135]
silica NPs	xylitol dehydrogenase	L-xylulose production	[136]
Carbon Nanotubes (CNTs)	Lipase	Biodiesel production	[137]
Poly amide amine nanoparticles	Hyaluronidase	Biocatalyst and therapeutics	[138]
Electrospun polyvinyl alcohol/Chitosan nanofibers	Urease	Drug delivery systems	[139]
Copper hybrid nanoflowers embedded with amine	Glucose oxidase	Antibacterial activity	[140]
Zinc ferrite NPs	Lipase	Removal of Methylene Blue dye, antibacterial activity	[141]
Chitosan nanoparticles	Cellulase	Bioethanol production from lignocellulosic biomass	[142]
Alginate beads	Laccase	Decolorization of textile dyes	[143]
Magnetic NPs	Lipase	Synthesis of biodiesel from waste cooking oil	[144]
Poly (lactic–co-glycolic acid) (PLGA) NPs	Catalase	Treatment of oxidative stress in neurodegenerative diseases	[145]
Polystyrene nanofiber (PSNF)	β-Galactosidase	Conversion of dairy waste into galacto-oligosaccharides (GOS)	[146]
Chitosan-coated iron oxide nanoparticles	peroxidase	Biosensor for phenolic compound detection	[147]
Fe_3_O_4_ magnetic nanoparticles	Trypsin	hydrolysis of bovine serum albumin and its application in the bovine milk	[148]
Chitosan/ZnO/Fe₂O₃ Nanocomposites	Catalase	Decomposition of hydrogen peroxide in industrial processes	[149]
Magnetic Nanoparticles	Pectinases	Hydrolysis of pectin in food processing and wastewater treatment	[150]
Green-synthesized ZnO–Fe₃O₄	Lipase	Application for the Synthesis of Lipophenols	[151]
Polydopamine-coated magnetic nanoparticles	Lipase	Not Applicable	[152]
Magnetic graphene oxide 3-Aminopropyl triethoxysilane Glutaraldehyde (MGO–AP–GA)	Lipase	Biodiesel production from Chlorella vulgaris bio-oil	[153]
Polymer nanoparticles encapsulated in poly ethylene glycol) (PEG)-based hydrogels	β-Galactosidase	Lactose hydrolysis in dairy industry and biosensing	[154]
Silica-coated magnetic nanoparticles functionalized with cysteine	Protease	Not Applicable	[155]
Cellulose-coated magnetic nanoparticles	α-Amylase	Starch degradation in food processing	[156]

## Recovery, potential risks and current challenges for the use of nanobiocatalysts in large-scale applications

The capacity to reutilize the biocatalyst is essential for the commercialization of nanobiocatalysts. The recovery process presents considerable hurdles, and the downstream processing necessary for their extraction post-reaction is exceedingly complex. The density difference between microcarriers or microbeads and the reaction media enable their easy separation. Integrating nanostructures with micro- or macrostructures can diminish the segregation of nanobiocatalysts. Nanostructured microparticles provide a nanoscale environment for enzymatic catalysis and are also recyclable for facile separation. Integrating magnetic technology with enzyme immobilization on nanocarriers improves the recoverability and reusability of nanobiocatalysts [157]. Nanobiocatalysts may be extracted from reaction fluid by engaging the magnetic field to draw magnetic nanocarriers towards magnetic sources. Nonetheless, the response medium may eliminate certain nanobiocatalysts due to negligible magnetic forces. Ngo et al*.* enhanced this technique by regulating the size of magnetic silica NPs [158]. Dissociation of the particles was performed to facilitate enzyme loading and reaction, and the particles were subsequently re-clustered to facilitate magnetic separation. Polymer-based nanocarriers may also be utilized with this magnetic technique. The magnetic particles were coated with polyaniline, which resulted in an exceptional magnetic response [159].

Identifying the source of emissions from production to disposal is essential for assessing the possible environmental issues associated with nanomaterials. Experimental data can be used in conjunction with mathematical models to assist in the examination of hazards and the determination of regulatory compliance. Nonetheless, the volume of data from the many stages of the life cycle of nanomaterials remains inadequate, requiring the collection of further information to improve the precision of the outcomes generated by these models [160]. The management of NMs throughout their functional lifespan presents an additional barrier. Minimal amounts may be emitted into the atmosphere throughout the production process. Respiratory exposure and changes in the air can constitute a significant source of toxicity, albeit representing a low percentage. Furthermore, increased atmospheric concentrations are expected in areas, where NMs are manufactured or discarded. Certain measures can be implemented to mitigate the likelihood of contamination, including the use of confined reactors and emission control devices during the synthesis process [161].

Nanotechnology is now crucial to the advancement and development of several research domains, including healthcare. Nanostructured materials have exhibited extraordinary potential as nanozymes or nano-enzyme supports. A wide range of nanocarriers, including those produced from polymers, silica, carbon, and metals, have been studied to encapsulate enzymes or multi-enzymes, resulting in the generation of functional and active nanobiocatalysts. The nanobiocatalysts exhibited enhanced enzyme activity and stability and significant potential for enzyme loading and recycling in bioprocesses. Commercialization continues to confront severe challenges.

- Cost reduction represents a significant barrier to extensive testing. The main costs go to enzyme preparation, immobilization, and nanocarrier production. Recent breakthroughs in protein engineering, fermentation, and purification technologies facilitate large-scale enzyme production; nonetheless, most nanocarriers are still produced at the laboratory scale.
- The viability of a large-scale method must be evidenced by the sustained operation of nanobiocatalysts over an extended period. The bulk of nanobiocatalysts produced in laboratory research are primarily evaluated for proof-of-concept, with just a limited fraction subjected to 10–15 rounds of testing. An evaluation of mechanical strength, permeability, activity, and stability is essential for prolonged operation.
- Bioprocess engineers play an important role in bringing bench-scale innovations to commercial applications. Most nanobiocatalysts are created by material scientists, and their potential has been emphasized. However, bioprocess engineers are responsible for making the promise a reality. Engineering issues should be addressed early in the construction process for NBC. Advanced multifunctional nanobiocatalysts may become commercially viable shortly thanks to the efforts of material scientists, bioprocessing engineers, and biochemists [157].
- The utilization of nanomaterials in industry is expanding, necessitating increased production rates. One of the primary issues with nanoparticles that cannot be disregarded is their toxicity, which is presently poorly understood and poses a significant threat to their industrial, household, and environmental applications. The extent to which nanoparticle-based compounds may induce cellular toxicity remains unknown. The scientific community should strive to bridge the information divide between the rapid development of nanomaterials and their potential in vivo toxicity. A comprehensive and systematic comprehension of the interactions between nanomaterials and cells, tissues, and proteins is necessary for the safe design and commercialization of nanotechnology [162].
- Finally, in specific situations, the general public's ignorance of the importance of nanotechnology hinders its extensive adoption across several sectors. Consequently, public acceptance of nanotechnology requires considerable work and comprehension of its importance [163].

## Conclusion

This review provides a comprehensive analysis of the advancements in enzyme immobilization on nanocarriers, focusing on the development and multifaceted applications of nanobiocatalysts. By integrating nanotechnology with biocatalysis, nanobiocatalysts address critical limitations of free enzymes, such as instability, high production costs, and challenges in recovery and reuse. The review highlights the distinctive properties of nanomaterials, including high surface area, tunable surface chemistry, and unique features such as magnetic or electrical conductivity, which make them exceptional platforms for enzyme immobilization. This review highlights their transformative impact across biomedical, environmental, and industrial domains, demonstrating their versatility in overcoming significant challenges.

Nanomaterials, such as magnetic nanoparticles, carbon-based nanostructures, porous silica, and metal–organic frameworks, serve as robust enzyme immobilization platforms, enhancing enzyme stability, activity, and recyclability. Advanced immobilization techniques, including physical adsorption, covalent bonding, entrapment, encapsulation, and cross-linking, enhance enzyme performance, with surface functionalization playing a pivotal role in tailoring enzyme–support interactions. Nanobiocatalysts have shown remarkable efficacy in applications, such as targeted drug delivery, thrombolytic therapy, wastewater treatment, pollutant degradation, biofuel production, food processing, and biosensing, underscoring their broad utility.

In biomedical contexts, nanobiocatalysts enable precise drug delivery and thrombolytic treatments with reduced side effects. In environmental applications, they offer sustainable solutions for wastewater treatment and contaminant degradation. Industrially, they enhance the efficiency of biofuel production and food processing, contributing to a greener sustainable economy. Despite these achievements, challenges remain, including cost-effective scaling, long-term stability, and addressing potential nanomaterial toxicity. Future research should explore emerging areas, such as energy storage, agriculture, cosmetics, and bioremediation of novel contaminants, such as microplastics and pharmaceutical residues. Furthermore, integrating nanobiocatalysts with cutting-edge technologies opens new frontiers in biosensing, personalized medicine, and tissue engineering. The continued evolution of nanobiocatalysts promises to drive sustainable innovation, shaping a technologically advanced and environmentally conscious future.

## Data Availability

The data presented in this study are available on request from the corresponding author.
